# Periodontal inflammation may amplify genitourinary vulnerability in perimenopause and postmenopause

**DOI:** 10.3389/fcimb.2026.1971575

**Published:** 2026-10-15

**Authors:** Yuxia Zhang, Jian Hua, Weisheng Li, Baoguo Xia, Chuyi Yang, Xiaoqun Hou, Weixin Chu

**Affiliations:** 1 Department of Gynecology, Qingdao Municipal Hospital, University of Health and Rehabilitation Sciences, Qingdao, China; 2 Department of Nursing, Shandong Medical College, Linyi, China; 3 Qingdao Municipal Hospital, University of Health and Rehabilitation Sciences, Qingdao, China

**Keywords:** genitourinary vulnerability, mucosal immunity, perimenopause, periodontal dysbiosis, recurrent UTI, systemic inflammation

## Abstract

During perimenopause and postmenopause, changes in estrogen exposure may alter mucosal barriers, microbial ecosystems, and inflammatory responsiveness across the oral and genitourinary tracts. These stages differ in their hormonal patterns, and much of the available evidence comes from postmenopausal women or experimental models of estrogen deficiency. In the oral cavity, altered salivary function, gingival immunity, epithelial integrity, and alveolar bone metabolism may increase the inflammatory potential of established biofilms without generating a uniform menopause-associated microbial signature. When periodontitis develops, disruption of the gingival barrier can promote repeated exposure to microbial products, cytokine spillover, hepatic acute-phase responses, endothelial activation, and myeloid activation, thereby adding to systemic inflammatory burden. In parallel, hypoestrogenism can weaken vaginal, urethral, and bladder epithelial resilience, reduce Lactobacillus-associated colonization resistance, and impair tissue repair. The association between hypoestrogenism and recurrent urinary tract infection is better supported than corresponding associations with bacterial vaginosis or vulvovaginal candidiasis. Genitourinary syndrome of menopause may cause overlapping symptoms and coexist with infection. In this narrative review, we propose an estrogen-sensitive oral-systemic-genitourinary axis in which periodontal inflammation may amplify genitourinary susceptibility through systemic inflammatory signals and possibly through changes in intestinal and vaginal ecosystems. Hypoestrogenism may provide a permissive background, while local microbial or inflammatory challenges contribute to clinical disease. This proposed interaction does not require direct bacterial dissemination to the genitourinary tract. Evidence supporting the individual components is stronger than direct human evidence for the complete pathway, and a contribution from persistent myeloid reprogramming remains unproven. Longitudinal multisite studies integrating hormonal trajectories, periodontal status, systemic immune markers, and microbiologically confirmed genitourinary outcomes, together with combined periodontal and local estrogen interventions, are required to test this hypothesis in perimenopausal and postmenopausal women.

## 1 The oral microbiome: ecological organization, homeostatic regulation, and disease-associated dysbiosis

Inter-organ axes describe functional communication between anatomically distinct tissues and their associated microbial communities. Such communication does not necessarily require microbial transfer between organs. Research on the gut-brain axis illustrates how microbial metabolites, immune responses, and neural and endocrine signals can mediate these interactions (The Microbiota-Gut-Brain Axis). The developing concept of a gut-vagina axis likewise includes proposed immune connections between intestinal and vaginal compartments (IgA and the gut-vagina axis). Here, we use the term axis to describe a proposed relationship in which periodontal inflammation may influence genitourinary susceptibility through circulating signals or intermediate microbial ecosystems, including the gut. This usage does not imply an established causal pathway.

### 1.1 Composition, spatial biogeography, and biofilm organization of the oral microbiome

The oral microbiome encompasses microorganisms occupying distinct oral niches, together with their genes, activities, and interactions with the host and local environment. Oral microbiota refers more specifically to the microorganisms themselves. Bacteria are currently the most extensively studied component, but ecological functions differ among species and strains within the same genus. An increase in genus-level abundance therefore does not, by itself, establish pathogenicity or causation (Baker et al., 2024). In addition to bacteria, fungi such as *Candida*, methanogenic archaea, bacteriophages, eukaryotic viruses, and protists participate in oral community interactions, although their functions are less well characterized (Diaz, 2021; Szafrański et al., 2021).

Oral communities are strongly shaped by their anatomical location. Teeth provide non-shedding hard surfaces that permit long-term microbial adhesion and the development of mature biofilms, whereas papillae and fissures on the dorsal tongue create numerous micron-scale hypoxic niches. Continuous epithelial shedding influences colonization of the buccal mucosa and palate, while oxygen availability, gingival crevicular fluid, and periodontal inflammation shape the gingival sulcus (Mark Welch et al., 2020; Wilbert et al., 2020). Saliva contains microorganisms shed from multiple oral surfaces and therefore provides a mixed community sample. It cannot fully substitute for samples taken directly from subgingival plaque, carious lesions, or root canals. The sampling site must therefore be considered when interpreting microbial associations with periodontal disease.

On tooth surfaces, dental plaque forms a structured multispecies biofilm embedded within a matrix of extracellular polysaccharides, proteins, lipids, and extracellular DNA. This matrix enhances the mechanical stability of the community, can limit antimicrobial penetration, and facilitates nutrient exchange and intercellular signaling (Cai and Kim, 2023; Hajishengallis et al., 2023).

During biofilm development, early colonizers adhere to the salivary pellicle, followed by recruitment of other microorganisms through coaggregation. *Fusobacterium nucleatum* can connect community members through adhesion and metabolic interactions, although its contribution depends on the strain and local microbial context (Sakanaka et al., 2022). Cross-feeding further supports community function. For example, Veillonella utilizes lactate produced by other bacteria, oxygen-consuming bacteria reduce local oxygen tension to support the survival of obligate anaerobes, and fungal hyphae provide attachment scaffolds for bacteria (Hajishengallis et al., 2023). These interactions support assessing the pathogenic potential of oral biofilms in terms of community activity and host responses, rather than the abundance of a single microorganism.

### 1.2 Host-microbial homeostasis and the ecological transition to dysbiosis

Oral health depends on a dynamic balance between resident microorganisms and host defenses. Many disease-associated microorganisms may be present at low abundance in healthy individuals, whereas some commensal organisms may become opportunistic pathogens when the local environment changes (Lamont et al., 2018). Homeostatic microbial communities suppress the expansion of exogenous pathogens by occupying adhesion sites, competing for nutrients, producing antimicrobial molecules, and modulating local immune responses. Bacterial metabolism of arginine and urea can help buffer local acidity, while nitrate reduction contributes to oral and systemic physiological functions (Eick and Lussi, 2021; Rosier et al., 2022).

Local barriers and immune defenses help maintain this balance. The oral epithelium, salivary antimicrobial proteins, secretory IgA, complement, macrophages, and neutrophils support microbial containment and limit invasion into deeper tissues (Williams et al., 2021). These defenses must control microbial growth without causing persistent tissue injury.

Sustained changes in the local environment can disturb this balance and favor dysbiosis. Dysbiosis is not synonymous with reduced microbial diversity. Rather, it refers to changes in microbial community composition, organization, or function associated with impaired host-microbial homeostasis. Indeed, the richness and diversity of diseased subgingival plaque in periodontitis may exceed those observed in health (Scannapieco and Dongari-Bagtzoglou, 2021). Dysbiosis describes an ecological condition; an altered microbial profile alone does not establish a diagnosis of periodontitis or quantify the accompanying tissue inflammation.

Microbial changes and inflammation can reinforce one another. Persistent plaque accumulation induces gingival inflammation, while increased gingival crevicular fluid, heme, peptides, and tissue degradation products provide nutrients that favor proteolytic anaerobes. The resulting community changes may further sustain inflammation and alter the subgingival environment (Hajishengallis et al., 2023).

The ecological plaque hypothesis and the polymicrobial synergy and dysbiosis model account for these interactions by considering microbial activity together with environmental conditions and host susceptibility. For the proposed oral-systemic-genitourinary axis, we therefore distinguish microbial dysbiosis from periodontal inflammatory burden, meaning the extent and intensity of inflammation in periodontal tissues. The latter is the exposure of primary interest in this review because the proposed systemic effects depend on the tissue response, rather than a change in microbial composition alone (**Figure 1**).

**Figure 1:**
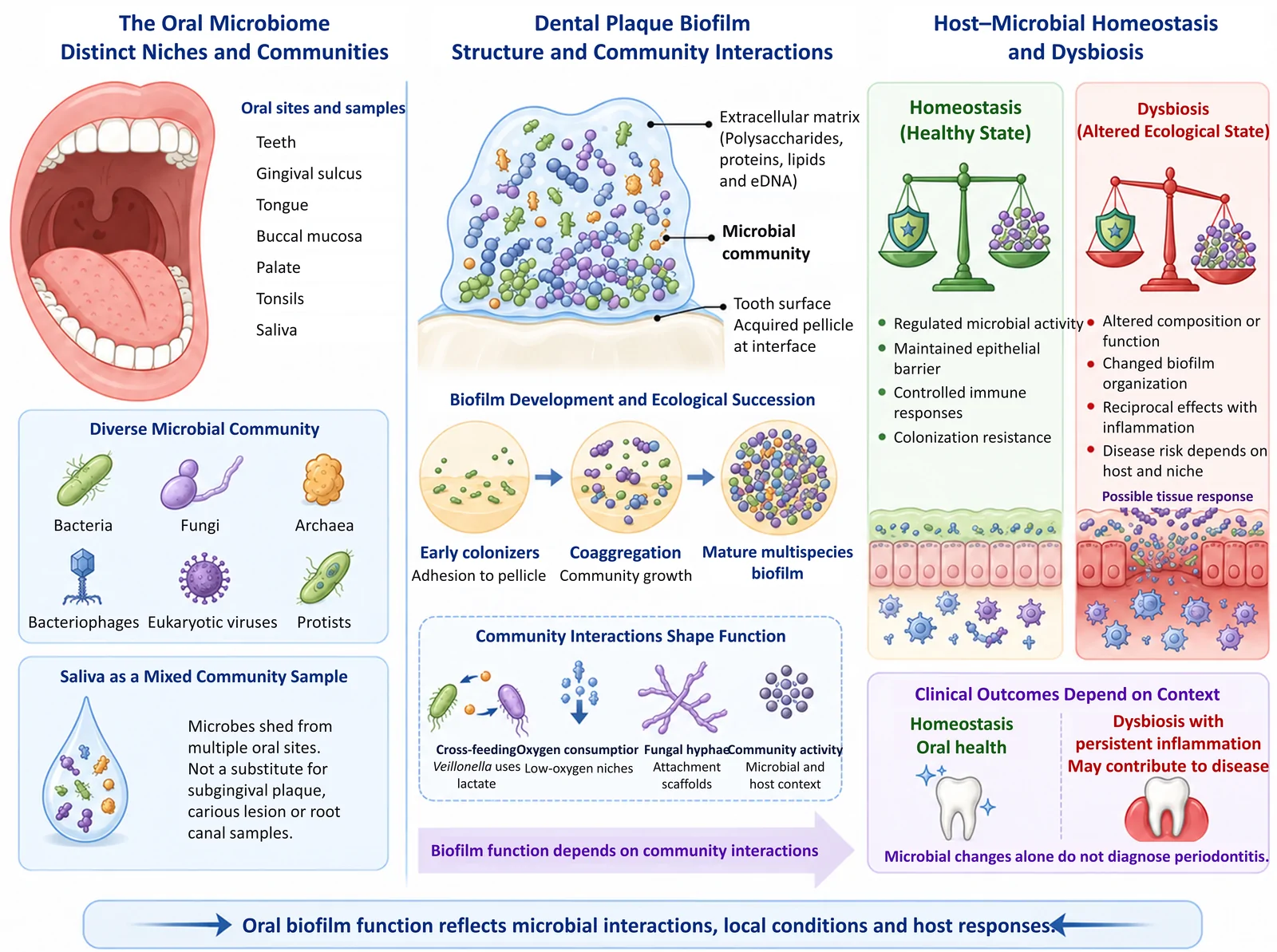
Oral microbial ecology and host homeostasis. Oral communities occupy anatomically distinct niches and interact with local host defenses. Dental plaque develops on the acquired pellicle of tooth surfaces as a multispecies biofilm embedded in an extracellular matrix. Coaggregation, nutrient exchange and local oxygen conditions influence community function. Saliva contains microorganisms shed from several surfaces and does not replace site-specific sampling. Homeostasis and dysbiosis describe ecological relationships between microorganisms, their environment and host responses. A microbial compositional change does not by itself diagnose periodontitis or establish systemic disease. The illustrations are schematic and do not represent measured relative abundances. eDNA, extracellular DNA.

### 1.3 Niche-specific oral diseases as distinct outcomes of ecological dysregulation

Differences in anatomy, nutrient availability, and host defenses help explain why oral dysbiosis produces different diseases at different sites. In dental caries, frequent sugar exposure favors acid-producing and acid-tolerant biofilms, causing demineralization to exceed remineralization. These biofilms can involve interactions between *Streptococcus mutans*, other bacteria, and fungi such as *Candida albicans* (Pitts et al., 2021; Wan et al., 2021).

Plaque-induced gingivitis is confined to the gingiva and presents with erythema, swelling, and bleeding on probing. It is generally reversible following plaque removal and does not inevitably progress to periodontitis (Abusleme et al., 2021). Moreover, individuals exposed to comparable plaque burdens may exhibit markedly different degrees of inflammation, demonstrating variation in host responses to plaque accumulation (Bamashmous et al., 2021).

Periodontitis involves inflammatory destruction of the tissues supporting the teeth. Its diagnosis requires clinical evidence of periodontal attachment loss, with radiographic bone loss informing assessment; microbial findings alone are insufficient (Papapanou et al., 2018). Tissue destruction reflects interactions between dysbiotic subgingival biofilms and a dysregulated host response (Darveau, 2010; Hajishengallis et al., 2023). Attachment and bone loss record accumulated damage and should be distinguished from current inflammation. Bleeding on probing and estimates such as periodontal inflamed surface area provide complementary information about inflammatory burden. The latter estimates the surface area of bleeding pocket epithelium (Nesse et al., 2008). These measures are relevant to testing the proposed systemic pathway, although they do not directly measure the release of inflammatory mediators into the circulation.

The traditional red complex, comprising *Porphyromonas gingivalis*, *Treponema denticola*, and *Tannerella forsythia*, remains representative of periodontitis-associated communities. However, sequencing studies show that periodontitis is associated with broader changes in subgingival community composition, diversity, and biomass (Abusleme et al., 2013). Experimental studies indicate that P. *gingivalis* may act as a low-abundance keystone pathogen that disrupts complement, Toll-like receptor, and neutrophil functions, thereby impairing microbial clearance and sustaining inflammation (Hajishengallis et al., 2011; Miralda and Uriarte, 2021).

Other oral diseases illustrate the importance of the local tissue environment. Persistent root canal infection involves multispecies communities within an anatomically complex space that can limit effective debridement and disinfection (Siqueira and Rôças, 2022). Oral candidiasis develops when conditions such as reduced salivary clearance, immunosuppression, diabetes, denture use, or antibiotic exposure permit *Candida* overgrowth and mucosal disease (Contaldo et al., 2023). Peri-implantitis occurs at a distinct interface where implant materials and surrounding tissues influence microbial colonization and inflammation (Kotsakis and Olmedo, 2021). These conditions should not be treated as interchangeable indicators of periodontal exposure. The present review focuses on inflammation in the tooth-supporting tissues as the proposed contributor to systemic inflammatory burden.

### 1.4 From microbial association to functional causality and systemic host context

Although numerous studies have identified microbial differences between healthy and diseased oral sites, such differences do not by themselves establish causality. An increase in a particular microorganism may represent a driver of disease, but it may also be a secondary consequence of inflammation, bleeding, tissue necrosis, dietary changes, medication use, or reduced salivary flow. An apparent increase in relative abundance may additionally result solely from a decline in other community members rather than from the actual expansion of the organism concerned (Ma et al., 2024).

Depending on the research question, absolute quantification, strain-level analysis, metagenomics, metatranscriptomics, metabolomics, spatial imaging, and host immune phenotyping can provide complementary information about microbial abundance, activity, organization, and interactions with host tissues (Baker et al., 2024). These approaches improve biological interpretation, but do not establish causality on their own.

Overall, the oral microbiome is a highly spatially organized and functionally redundant ecosystem that continuously interacts with host immunity. Oral diseases should not be uniformly interpreted as the invasion of exogenous pathogens. More often, they represent distinct pathological outcomes arising from the concurrent disruption of environmental conditions, microbial communities, and host responses within specific ecological niches. Periodontitis, in particular, extends beyond local changes in microbial composition and involves long-term remodeling of the epithelial barrier, innate immunity, and chronic inflammatory networks.

These observations also indicate that oral microbial homeostasis cannot be understood independently of the broader physiological condition of the host. Mucosal barrier integrity, immune responsiveness, salivary function, inflammatory regulation, and tissue repair capacity are all influenced by systemic physiological states. It is therefore necessary to return to the female life course before considering how the oral ecosystem may contribute to extraoral disease susceptibility and to examine perimenopause as a major systemic transition capable of reshaping both oral microbial ecology and host immune homeostasis.

## 2 Endocrine and immune changes in perimenopause and postmenopause

### 2.1 Reproductive staging and changes in endocrine and immune function

This review covers perimenopause and postmenopause, using the STRAW + 10 framework to distinguish reproductive stages. The menopause transition begins with persistent changes in menstrual cyclicity and ends at the final menstrual period. Perimenopause includes this transition and the following 12 months. Natural menopause is confirmed retrospectively after 12 months of amenorrhea without another cause. Postmenopause refers to the period after the final menstrual period and therefore overlaps with the final year of perimenopause. Hormonal variability during the transition gradually gives way to more sustained hypoestrogenism after menopause (Harlow et al., 2012; Santoro et al., 2021). These definitions provide the framework for interpreting the evidence, while the populations and staging criteria reported in individual studies remain relevant. Studies of postmenopausal women are not treated as direct evidence for perimenopause, and experimental estrogen deficiency is considered separately from natural reproductive aging.

Changes in estrogen exposure can affect a wide range of estrogen-responsive peripheral tissues, including the oral mucosa, periodontal tissues, vagina, and urinary tract. Attenuated estrogen signaling may impair epithelial renewal, tissue barrier integrity, local immune defense, and the maintenance of microbial habitats, although the effects vary with tissue, hormonal exposure, and host condition (Yang et al., 2024; Nieto et al., 2025).

Inflammatory changes are also heterogeneous. In the longitudinal Study of Women’s Health Across the Nation, trajectories of high-sensitivity C-reactive protein and IL-6 differed among women. Some groups showed increases around the final menstrual period, whereas others had stable or declining concentrations. Premenopausal overweight or obesity and race or ethnicity were associated with different trajectory patterns (El Khoudary et al., 2025). Neither perimenopause nor postmenopause should therefore be described as a uniformly proinflammatory state. Because estrogen contributes to the regulation of both innate and adaptive immune homeostasis, its fluctuation and subsequent decline may modify immune-cell function, but reduced estrogen exposure should not be assumed to produce the same inflammatory response in every tissue or individual (Yang et al., 2024).

### 2.2 Coordinated remodeling of oral and genitourinary mucosal ecosystems

Perimenopause and postmenopause provide related hormonal contexts in which oral and genitourinary tissues may undergo changes. Although the oral cavity and genitourinary tract differ substantially in their microbial composition, anatomical structure, and local immune environment, both are influenced by estrogen signaling, epithelial barrier integrity, the quantity and composition of local secretions, and the magnitude of host inflammatory responses (Nieto et al., 2025). As hypoestrogenism develops, the oral cavity may exhibit reduced salivary secretion, mucosal dryness, impaired periodontal homeostasis, and alterations in microbial community structure. In parallel, the genitourinary tract may undergo epithelial thinning, reduced glycogen availability, less frequent *Lactobacillus* dominance, and reduced resistance to microbial colonization and infection (Clark and Goetsch, 2024; Labunet et al., 2025). These changes need not occur together or to the same extent in an individual woman. Much of the clinical evidence concerns postmenopausal populations, and the timing of changes across the transition remains less well defined.

Shared hormonal influences on the two compartments do not establish a causal relationship between them. The hypothesis considered here is that periodontal inflammation may add to systemic inflammatory burden and modify the response of susceptible genitourinary tissues to local challenges. Its relevance may depend on reproductive stage, hormone therapy, metabolic health, and the extent of periodontal inflammation. By considering both perimenopause and postmenopause, this review examines that possibility across a broader clinical population without assuming that the underlying hormonal or immune conditions are identical (**Figure 2**).

**Figure 2:**
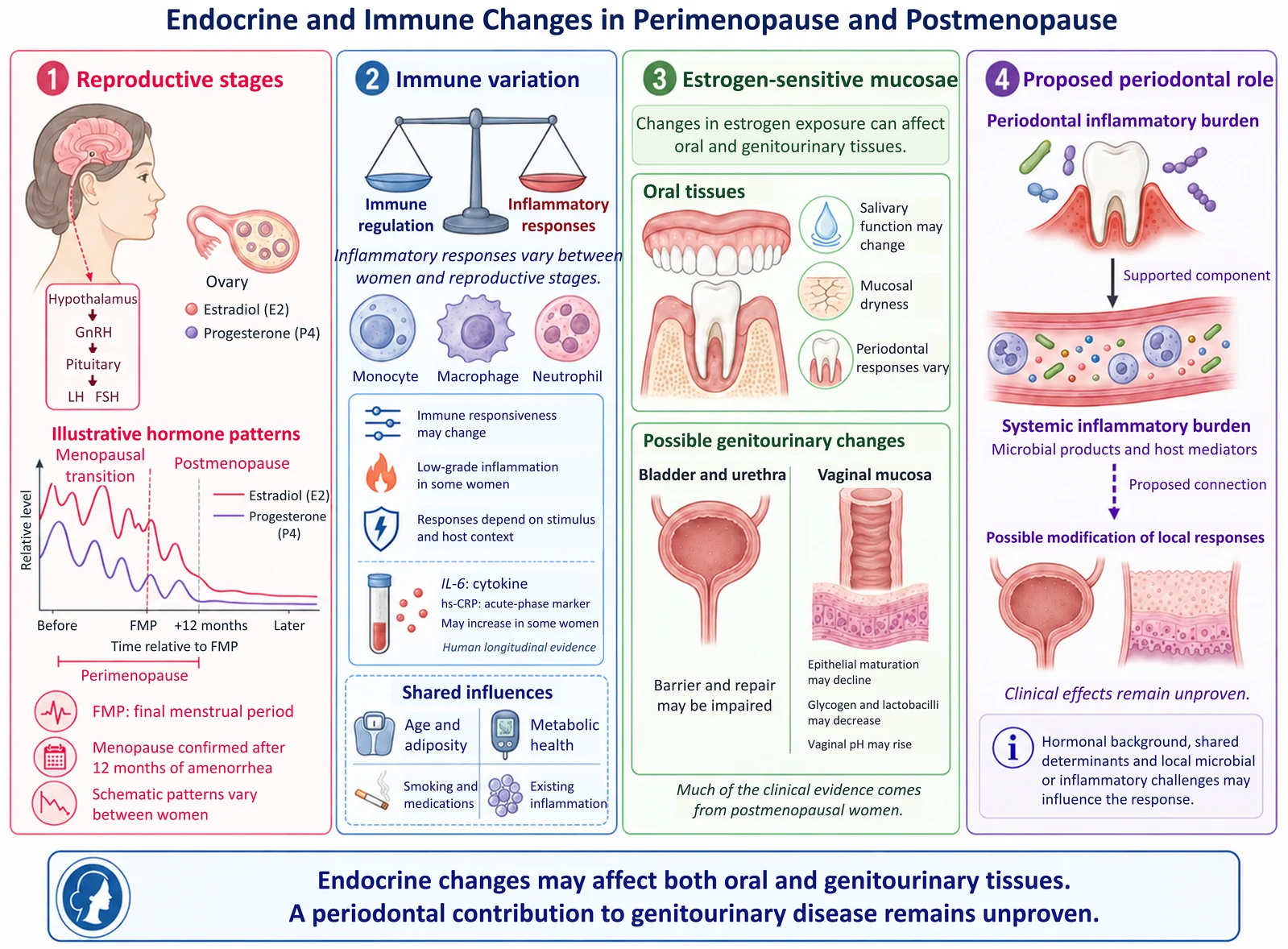
Endocrine and immune changes in perimenopause and postmenopause. Schematic hormone patterns illustrate variability during the menopausal transition and generally lower estrogen exposure after the final menstrual period. Stage definitions follow Section 2.1; the curves are illustrative and do not prescribe an age or trajectory for an individual woman. Oral and genitourinary tissues can respond differently to estrogen exposure, and systemic inflammatory responses vary with host characteristics. Vaginal epithelial maturation and microbial ecology are distinguished from lower urinary barrier function. Much of the clinical mucosal evidence comes from postmenopausal women. The solid periodontal-systemic arrow identifies a supported component relationship, whereas the dashed connection to genitourinary susceptibility represents the proposed extension. The figure does not establish clinical benefit from periodontal treatment. E2, estradiol; FMP, final menstrual period; hs-CRP, high-sensitivity C-reactive protein; IL-6, interleukin-6.

## 3 Oral microbial ecology in perimenopause and postmenopause

### 3.1 Hormonal remodeling of salivary and mucosal ecological niches

Hormonal changes during perimenopause and postmenopause may influence oral microbial communities by altering saliva, mucosal tissues, periodontal immunity, and bone metabolism. However, the available literature does not establish a consistent sequence of microbial changes across these stages (Tramice et al., 2022; Lopes Cardoso et al., 2026). Much of the clinical evidence comes from comparisons between premenopausal and postmenopausal women. These studies describe differences between groups but cannot determine when those differences developed or separate reproductive aging from all other age-related influences.

The sensitivity of the oral cavity to hormonal changes may partly reflect the expression of sex hormone receptors in the salivary glands, oral mucosa, and gingival tissues. Estrogen signaling contributes to epithelial maturation, tissue hydration, vascular regulation, collagen metabolism, and glandular secretory function (Ciesielska et al., 2021; Shrivastava, 2024). Changes in these host functions provide plausible routes through which hormonal exposure could influence established microbial communities. Whether these processes occur differently during perimenopause and postmenopause remains incompletely resolved.

Saliva supports microbial clearance, buffering, and local defense. Clinical reports describe xerostomia, oral burning, altered taste, and changes in salivary function in women during reproductive aging, particularly after menopause (Tramice et al., 2022; Labunet et al., 2025). Reduced salivary flow can impair clearance and alter the conditions in which oral biofilms develop. However, the presence of these symptoms does not establish a hormonal cause.

Subjective xerostomia should also be distinguished from objectively measured hyposalivation and from microbial dysbiosis. Oral dryness can occur without a corresponding reduction in measured flow, while salivary biochemical differences may be detected without substantial changes in bacterial composition (Tramice et al., 2022; Labunet et al., 2025). Symptoms, flow measurements, and microbial findings therefore provide related but distinct information.

### 3.2 Salivary biochemical differences and microbial composition

The AMICA study compared 19 menstruating women with 20 postmenopausal women. It found no significant overall difference in salivary bacterial composition, but identified differences in metabolites, glycoside hydrolase activities, and lipid mediators (Tramice et al., 2022). These findings show that biochemical differences can coexist with limited detectable taxonomic differences. They do not establish that functional changes precede microbial restructuring, because participants were not followed through the menopause transition. Nor do salivary metabolite measurements alone distinguish microbial production from host or dietary contributions.

A cross-sectional study published in 2024 examined 30 women aged 40 to 65 years, classified by the investigators as premenopausal or postmenopausal. Samples were collected from the buccal mucosa, tongue dorsum, supragingival plaque, and subgingival plaque. Salivary estrone and estradiol showed different associations with microbial profiles across these sites. Estrone was associated with tongue microbial diversity and enrichment of *Porphyromonas*, whereas estradiol showed positive associations with *Streptococcus* and *Lactobacillus* and negative associations with *Fusobacterium* and *Prevotella* (J Rus et al., 2026). These findings are exploratory. The small sample and cross-sectional design do not establish hormonal effects on individual taxa, and genus-level associations do not determine microbial pathogenicity.

In the Buffalo OsteoPerio study, a cross-sectional analysis of 1,270 postmenopausal women found differences in subgingival diversity and 18 taxa across categories of menopausal hormone therapy use. Differences between current and never users were largely unchanged after adjustment for age, body mass index, and oral hygiene (Soliman et al., 2022). This association does not establish that hormone therapy directly altered the microbiome. Together, these studies support measuring hormonal exposure, salivary biochemistry, and microbial composition separately, rather than assuming that one measurement adequately represents the others.

### 3.3 Hormonal influences on periodontal tissues and bone

Hormonal exposure may also influence the tissue response to plaque. Reviews of clinical and experimental evidence describe possible effects on gingival integrity, collagen turnover, immune responses, and periodontal outcomes, although the evidence does not define a consistent mechanism across reproductive stages (Lopes Cardoso et al., 2026; Martín FCS et al., 2026). These processes could influence the inflammatory consequences of an existing biofilm without requiring a distinct menopause-associated microbial community.

A 2025 study comparing the subgingival microbiota of premenopausal and postmenopausal women identified more pronounced clinical attachment loss in postmenopausal participants. However, menopausal status was not associated with an overall dysbiotic shift, and lower estradiol levels did not consistently correspond to differences in bacterial abundance (Yakar et al., 2025). This observation separates periodontal tissue damage from the measured microbial profile. It does not establish that estrogen deficiency caused the greater attachment loss, because chronological age and accumulated disease exposure may also contribute.

Postmenopausal osteoporosis and periodontal disease have also been associated in clinical studies. Experimental mechanisms involving estrogen deficiency, RANKL signaling, and osteoclast activity provide a possible explanation for interactions between systemic bone loss and periodontal destruction (Jayusman et al., 2023; Qi et al., 2023). However, these associations do not demonstrate that systemic bone loss causes oral dysbiosis. Any effect on microbial ecology through changes in periodontal anatomy remains an indirect interpretation.

The hormone therapy findings described in Section 3.2 likewise provide observational evidence rather than proof of microbial restoration (Soliman et al., 2022). Users and non-users may differ in healthcare access, health behavior, metabolic status, and treatment history. These differences should be considered before attributing microbial or periodontal findings to hormone therapy itself.

### 3.4 Contributing factors, evidence limitations, and an integrated ecological model

Concurrent disease can complicate the interpretation of hormonal associations. In a study of patients with COVID-19, postmenopausal women had higher salivary IFN-γ than the comparison groups, while increases in several other cytokines were not statistically significant. Microbial differences were also associated with oral health status (Bayardo-González et al., 2024). These findings cannot isolate the contribution of menopausal status from the effects of infection and associated clinical differences, and should not be generalized to otherwise healthy women.

Stress and metabolic conditions provide additional possible influences. Experimental observations summarized in the literature indicate that cortisol can alter transcriptional activity in oral microbial communities (Nieto et al., 2025). Such observations support a possible biological pathway but do not establish that stress-related microbial changes account for differences between perimenopausal and postmenopausal women.

Medication exposure is an important confounder because several commonly prescribed drugs, including some antidepressants, sedatives, and antihypertensives, can reduce salivary secretion (Fortuna et al., 2023). Oral microbial composition may also be influenced by smoking, diet, alcohol consumption, oral hygiene, denture use, tooth loss, antibiotic exposure, diabetes, and previous periodontal treatment (Monson et al., 2022; Galvin et al., 2023). Interpretation should therefore account for chronological age, reproductive stage, medication and hormone therapy exposure, periodontal status, and relevant health behaviors. The role of each variable should be considered in relation to the specific association being examined.

Evidence linking reproductive stage to oral fungal disease is also limited. Xerostomia and hyposalivation are associated with oral candidiasis (Molek et al., 2022). However, fungal detection does not necessarily explain oral symptoms. A study of patients with burning mouth syndrome found no significant association between Candida infection and burning severity. These findings support distinguishing colonization, clinically apparent candidiasis, and symptoms, without attributing them collectively to estrogen decline (Jankovskis and Selga, 2022).

Prospective evidence provides a different perspective. In a cohort of postmenopausal women, baseline subgingival microbial features were associated with alveolar bone loss measured five years later (LaMonte et al., 2021). This temporal association is relevant to periodontal prognosis but does not establish that menopause caused the microbial differences or identify a functional mechanism.

Overall, the evidence is consistent with hormonal exposure acting alongside local inflammation, metabolic health, medications, and behavior to influence oral ecology and periodontal susceptibility (Sedghi et al., 2021). A reproducible microbial signature specific to perimenopause or postmenopause has not been established across studies. The relevance of these oral findings to the proposed genitourinary pathway therefore depends on whether they are accompanied by clinically meaningful periodontal inflammation (**Figure 3**).

**Figure 3:**
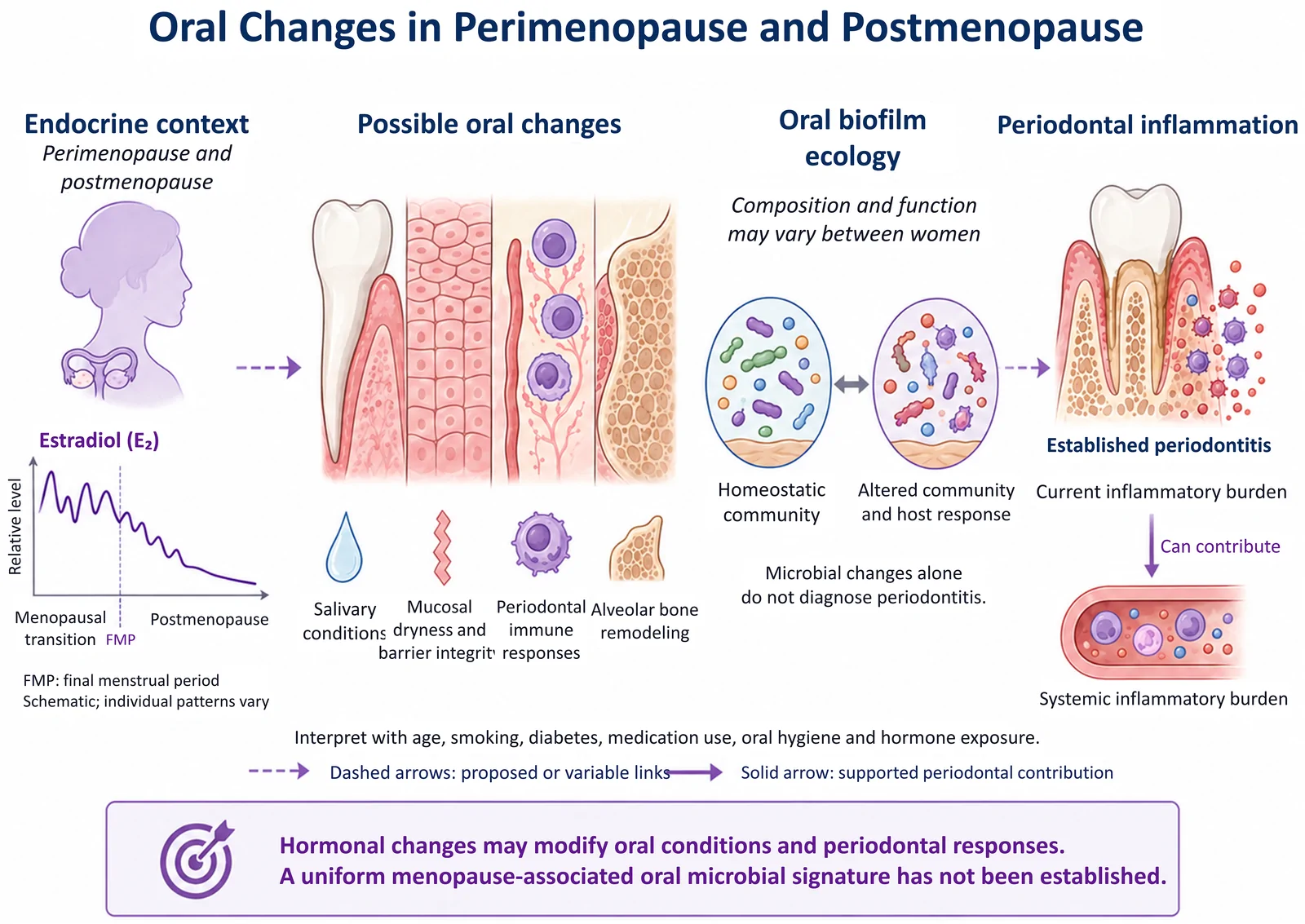
Oral changes in perimenopause and postmenopause. Changes in estrogen exposure may modify salivary conditions, mucosal integrity, periodontal immune responses and alveolar bone remodeling. These features vary among women and require interpretation alongside age, medication use, smoking, diabetes, oral hygiene and hormone exposure. Biofilm composition and activity do not identify an inevitable sequence from hormonal change to periodontal disease, and the figure does not imply that functional changes precede compositional changes. Established periodontitis and its current inflammatory burden are distinguished from microbial observations alone. Dashed arrows denote proposed or variable links; the solid periodontal-systemic arrow represents a supported component. The hormone curve is schematic. A uniform menopause-associated oral microbial signature has not been established. E2, estradiol; FMP, final menstrual period.

## 4 Periodontal inflammation as a contributor to systemic inflammatory burden

### 4.1 Periodontal barrier disruption and the systemic dissemination of microbial signals

An intact oral epithelium and local immune defenses limit microbial access to underlying tissues. Transient bacteremia can nevertheless occur during daily activities or dental procedures and is generally cleared by host defenses. In periodontitis, persistent inflammation and disruption of the pocket epithelium provide a plausible basis for more frequent exposure to microbial products and host-derived inflammatory mediators (Hajishengallis and Chavakis, 2021; Vitkov et al., 2023). The exposure relevant to the proposed systemic pathway is therefore inflammation in diseased periodontal tissues, rather than microbial compositional change alone.

Periodontal tissues are a key interface between subgingival biofilms and the circulation. Junctional epithelium and neutrophil surveillance normally contain microorganisms, whereas experimental evidence shows that excessive neutrophil elastase can damage epithelial adhesion molecules and impair barrier integrity (Hiyoshi et al., 2022). In periodontitis, ulcerated pocket epithelium exposes inflamed tissue and capillaries to bacteria and inflammatory mediators (Hajishengallis and Chavakis, 2021). Human studies document transient bacteremia after procedures and activities including extraction, scaling, toothbrushing, and chewing (Martins et al., 2024). The contribution of these episodes to persistent inflammation depends on exposure and host response and cannot be inferred from bacteremia alone. Lipopolysaccharides, proteases, nucleic acids, and bacterial extracellular vesicles may also disseminate and activate innate immune pathways (Martínez-García and Hernández-Lemus, 2021; Ha et al., 2023). Much of the evidence for the distant effects of these products is experimental. Detection of bacterial DNA does not establish viable bacterial dissemination, persistent colonization, or an oral source without additional evidence.

### 4.2 Inflammatory spillover, hepatic amplification, and evidence from periodontal treatment

Periodontal lesions may promote systemic inflammation through both microbial dissemination and the release of host-derived mediators. Inflamed gingiva contains elevated IL-1β, IL-6, TNF, IL-17, chemokines, prostaglandins, and matrix-degrading enzymes, reflecting sustained activation of epithelial, myeloid, and stromal cells (Plemmenos et al., 2021). IL-6 provides a plausible link to hepatic amplification because it activates hepatocyte STAT3 signaling and induces C-reactive protein, serum amyloid A, fibrinogen, and other acute-phase proteins (Rose-John, 2022). IL-6 is an upstream cytokine, whereas CRP is an acute-phase protein. Neither is specific to periodontal inflammation.

In a cross-sectional study of older Japanese adults, periodontal inflamed surface area, an estimate of the surface area of bleeding pocket epithelium, was associated with higher hs-CRP after adjustment for measured covariates (Miki et al., 2021). This association does not exclude residual confounding or establish the direction of the relationship. A systematic review concluded that periodontal treatment can improve some systemic inflammatory and cardiometabolic measures, although heterogeneity and limited long-term data constrain interpretation (Orlandi et al., 2022). In patients with type 2 diabetes, a recent meta-analysis of randomized trials likewise reported reduced hs-CRP after periodontal therapy (da Silva Barbirato et al., 2024). These results support an effect on measured inflammatory markers, but do not establish a benefit for genitourinary outcomes.

Sampling time is important when interpreting treatment effects. A randomized trial in patients with type 2 diabetes found a greater immediate acute-phase response after full-mouth treatment than after treatment delivered by quadrant (Graziani et al., 2023). Meta-analytic evidence also indicates reductions in several inflammatory markers during subsequent follow-up, although the magnitude and timing vary among markers and studies (Cardisciani et al., 2025). Blood-sampling time should therefore be standardized to distinguish transient treatment-induced inflammation from sustained systemic improvement.

### 4.3 Vascular effects, experimental myeloid training, and the oral-gut route

Experimental and clinical evidence suggests that periodontal inflammation may affect vascular and hematopoietic compartments. Microbial ligands and inflammatory mediators can promote endothelial activation and leukocyte recruitment (Li et al., 2022). Periodontitis has also been associated with altered platelet transcriptional states and coagulation-related pathways (Lee et al., 2023). These findings are not specific to periodontitis, and their interpretation is complicated by smoking, diabetes, obesity, and other inflammatory conditions (Ge et al., 2024).

Persistent changes in myeloid responsiveness have been demonstrated in experimental systems. In mouse models, periodontitis induced IL-1-dependent reprogramming of hematopoietic stem and progenitor cells, and bone marrow transfer increased susceptibility to inflammatory arthritis in recipient animals (Li et al., 2022). *In vitro* exposure of human bone marrow progenitors to IL-1β produced monocyte descendants with increased cytokine responses to subsequent stimulation and altered metabolic and transcriptional activity (Flores-Gomez et al., 2024). These findings support the biological possibility of persistent myeloid changes, but do not demonstrate this mechanism in perimenopausal or postmenopausal women with recurrent genitourinary disease. Trained immunity refers to a lasting change in innate immune responsiveness associated with metabolic or epigenetic reprogramming. CRP elevation alone is a nonspecific inflammatory finding; cellular activation or enhanced responsiveness during ongoing stimulation does not establish immune memory (Netea and Joosten, 2024). We therefore use myeloid activation or priming for findings that do not demonstrate persistence beyond the initiating stimulus.

The oral-gut route provides an additional, context-dependent pathway. In susceptible mice, periodontitis-associated oral bacteria and orally primed Th17 cells contributed to intestinal inflammation (Kitamoto et al., 2020). Transplantation of salivary microbiota from patients with severe periodontitis altered murine gut communities, impaired intestinal barrier-related measures, and induced low-grade intestinal inflammation (Bao et al., 2022). However, swallowing oral bacteria does not imply stable intestinal colonization. Intestinal expansion depends on both microbial exposure and the recipient ecosystem, including its colonization resistance (Kitamoto and Kamada, 2022).

These experiments identify the gut as a possible intermediary between oral disease and distant tissue responses. Within the proposed model, intestinal ecological or immune changes could influence vaginal or periurethral conditions and, in turn, bladder susceptibility. This extension was not tested in the cited oral-gut experiments. Human intervention evidence is currently more direct for changes in circulating inflammatory markers after periodontal treatment than for a complete intestinally mediated pathway to genitourinary disease. The two routes may coexist, but their relative contributions remain unknown.

### 4.4 Periodontitis as a contributor to systemic inflammation: evidence and causal limits

Longitudinal studies provide evidence of systemic associations, although their outcomes are largely outside the genitourinary tract. In a 13-year prospective cohort, periodontitis and systemic inflammatory markers showed independent associations and interaction on the additive scale with all-cause and cardiovascular mortality (Pink et al., 2023). A five-year longitudinal study also found that CRP and leukocyte counts statistically accounted for part of the association between periodontitis and incident hypertension (Torrungruang et al., 2024). Such mediation analyses are consistent with an inflammatory pathway but do not prove its mechanism.

Periodontitis shares major determinants with cardiovascular, metabolic, and immune-mediated diseases, including smoking, obesity, diabetes, age, diet, and socioeconomic disadvantage (Larvin et al., 2023). Longitudinal evidence concerning periodontitis and inflammatory bowel disease also suggests that associations can operate in both directions (Wang et al., 2024). Shared causes and reverse effects must therefore be considered alongside a possible contribution from periodontal inflammation.

Periodontal treatment can reduce serum IL-6 and hs-CRP in systemically healthy patients (Matsuda et al., 2024), but biomarker improvement does not establish prevention of cardiovascular or other extraoral clinical events (Kim et al., 2024). The available evidence supports periodontal inflammation as a potentially modifiable contributor to systemic inflammatory burden. Whether reducing that burden improves genitourinary outcomes in perimenopausal or postmenopausal women remains unproven. This distinction separates evidence for a systemic effect of periodontal disease from evidence for the complete axis proposed in this review (**Figure 4**).

**Figure 4:**
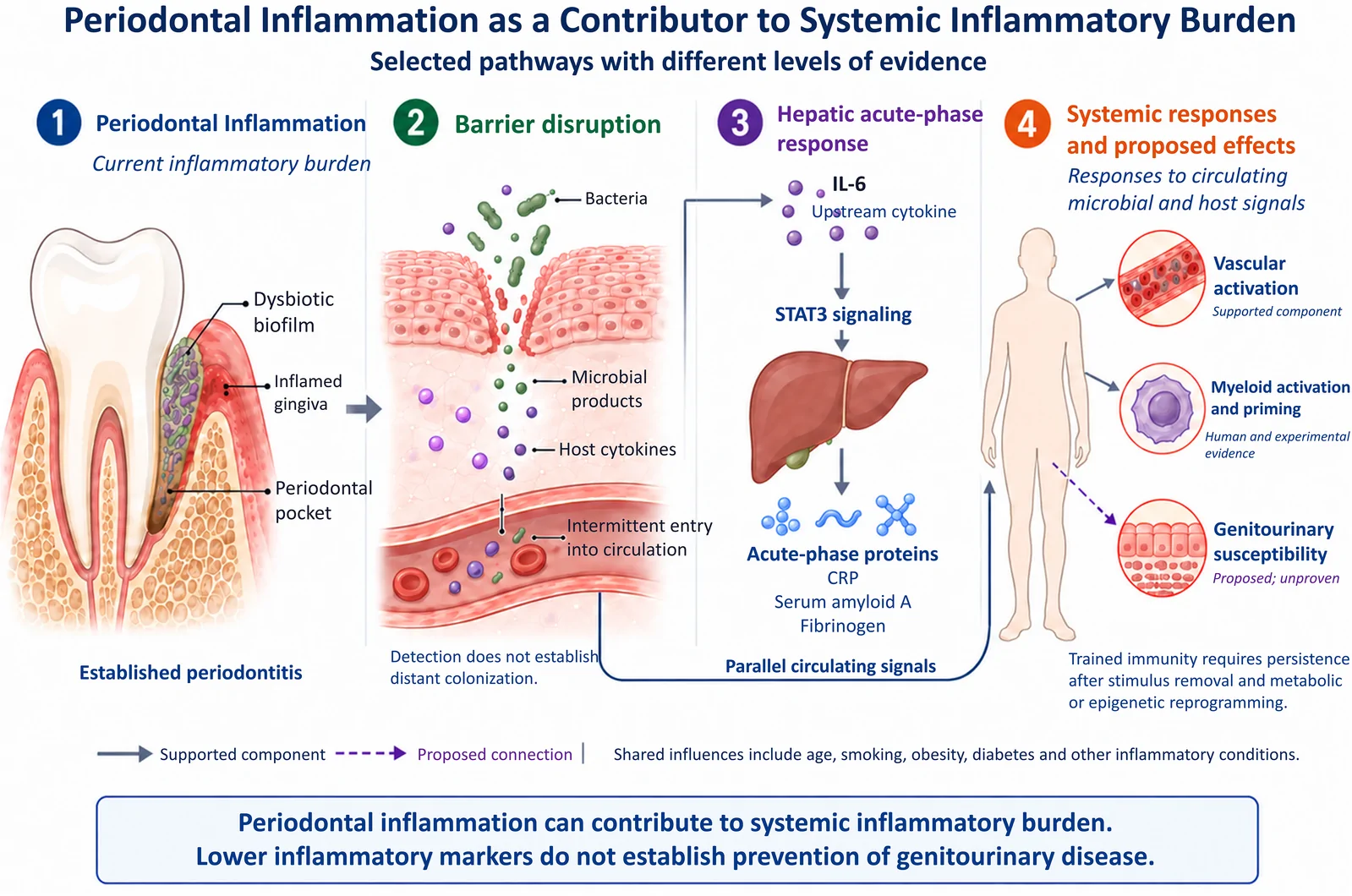
Periodontal inflammation and systemic inflammatory burden. Inflamed periodontal tissues may permit intermittent entry of microorganisms, microbial products and host mediators into the circulation. IL-6 can stimulate hepatocyte STAT3 signaling and production of acute-phase proteins, including CRP, serum amyloid A and fibrinogen. Parallel circulating signals may also influence vascular and myeloid responses without requiring mediation by these hepatic proteins. Myeloid activation and priming are distinguished from trained immunity, which requires persistent functional change after stimulus removal with metabolic or epigenetic evidence. Solid arrows indicate supported components; the dashed genitourinary connection is hypothetical. Evidence varies across human studies and experimental models. Detection of microbial material does not establish distant colonization, and lower inflammatory markers do not establish prevention of genitourinary disease. CRP, C-reactive protein; IL-6, interleukin-6; STAT3, signal transducer and activator of transcription 3.

## 5 Genitourinary vulnerability in perimenopause and postmenopause: distinct clinical phenotypes

In this review, genitourinary vulnerability describes a proposed susceptibility to persistent GSM symptoms, recurrent symptomatic urinary infection, and clinically defined vaginal disorders. It is not a standardized diagnosis or a composite clinical endpoint. GSM symptom burden, culture-confirmed urinary recurrences, and individual vaginal diagnoses should therefore be assessed separately. Microbial composition, vaginal pH, and inflammatory markers may help explain these outcomes but cannot substitute for them.

### 5.1 Hypoestrogenism, barrier deterioration, and genitourinary ecological instability

Recurrent dryness, burning, pruritus, urgency, frequency, dysuria, and abnormal discharge during perimenopause and postmenopause have several possible causes. These symptoms may arise from genitourinary syndrome of menopause (GSM), a clinical syndrome encompassing estrogen-related vulvovaginal and lower urinary tract symptoms and signs, recurrent urinary tract infection, bacterial vaginosis, vulvovaginal candidiasis, or other vaginitides (Powell et al., 2024; Kaufman et al., 2025). GSM can coexist with infection, and the contribution of each condition requires clinical assessment. Symptoms alone do not establish recurrent infection; testing should be selected according to the suspected urinary or vaginal disorder.

Changes in estrogen exposure during perimenopause and postmenopause affect the vagina, vulva, urethra, and bladder. Reduced estrogenic signaling impairs epithelial maturation, lubrication, elasticity, perfusion, and repair, producing thinner, injury-prone mucosa (Sarmento et al., 2021). Unlike many vasomotor symptoms, GSM often persists after menopause and may progressively compromise local barrier function.

Hormonal change also remodels the vaginal and periurethral microbial environment. Reduced epithelial maturation and glycogen availability can weaken Lactobacillus dominance, decrease lactic-acid production, raise vaginal pH, and increase community heterogeneity. In an analysis of postmenopausal participants in SWAN, vaginal atrophy markers were associated with non-Lactobacillus-dominant communities, whereas estrogen exposure and higher estrone or estradiol were associated with Lactobacillus crispatus dominance (Waetjen et al., 2023). Cross-sectional metagenomic profiling has also identified associations of rUTI history and estrogen exposure with microbial composition and functional potential in postmenopausal women (Neugent et al., 2022). These findings do not establish the sequence of microbial changes during perimenopause or show that a particular community necessarily causes symptoms.

Previous infection may independently alter subsequent tissue responses. Experiments using urothelial progenitor cells derived from previously infected mice demonstrated persistent epigenetic changes and altered responses to further infection (Russell et al., 2023). This is evidence of local epithelial memory in an experimental model. It does not establish that menopause or periodontal inflammation produces the same changes in women, nor does it explain persistent symptoms without further clinical assessment.

### 5.2 Recurrent urinary tract infection and the gut-vagina-bladder continuum

Recurrent urinary tract infection (rUTI), used here to refer to recurrent symptomatic cystitis, is commonly defined as at least two symptomatic episodes within 6 months or three within 12 months (Anger et al., 2019). The 2025 AUA/CUA/SUFU guideline emphasizes symptoms, pyuria, and uropathogenic bacteria, with urinalysis and urine culture obtained before treatment of each suspected recurrence. Molecular detection alone does not establish symptomatic infection and should not be treated as interchangeable with culture. The guideline also recommends vaginal estrogen for perimenopausal and postmenopausal women with rUTI when there is no contraindication to its use (Ackerman et al., 2026).

Among the infectious outcomes considered in this review, rUTI has the strongest clinical support for a contribution from hypoestrogenism, particularly in postmenopausal women. Hypoestrogenism is not sufficient for recurrence, but it can alter epithelial integrity and vaginal colonization resistance, potentially increasing periurethral exposure to uropathogens. Between episodes, postmenopausal women with rUTI may exhibit urinary microbial communities distinct from controls (Vaughan et al., 2021).

Local estrogen therapy provides interventional evidence relevant to this susceptibility background. A randomized trial in postmenopausal women found that fewer participants receiving vaginal estrogen experienced a UTI during follow-up than those receiving placebo (Ferrante et al., 2021). Vaginal estrogen can also increase urogenital *Lactobacillus*, particularly *L. crispatus*, although microbial restoration does not always predict clinical success (Jung et al., 2022). These findings support a preventive role for vaginal estrogen but do not establish that its clinical benefit is mediated entirely by microbial changes.

Recurrence also involves extraurinary reservoirs. A year-long study of women with and without a history of rUTI found lower intestinal microbial richness and fewer butyrate-producing bacteria in the rUTI group. However, intestinal and urinary *Escherichia coli* populations were comparable between groups in relative abundance and phylogroup (Worby et al., 2022). These findings support an association involving the intestinal ecosystem and host responses, rather than a general explanation based on intestinal overgrowth of *E. coli.* They do not isolate an effect of menopausal stage.

A separate cross-sectional study of 62 postmenopausal women reported vaginal differences in *Lactobacillus* species, a greater burden of antimicrobial-resistance markers in women with rUTI, and concordance between rectal organisms and urogenital findings. The design could not establish transmission direction, and organism or resistance concordance did not demonstrate strain identity (Horseman et al., 2026).

Together, these observations support considering the intestinal reservoir, vaginal colonization resistance, periurethral exposure, and ascending bladder infection as connected processes. Antibiotic exposure can modify microbial communities and select resistant organisms, while local estrogenic status influences the vaginal environment. The vaginal compartment may therefore affect urinary susceptibility through its role in limiting or permitting uropathogen carriage. Its contribution may differ between women and between episodes.

Experimental studies of intracellular infection provide a further perspective on recurrence. Work on uropathogenic *E. coli* (UPEC) and Nur77 shows that urothelial defenses can influence intracellular bacterial infection (Collins et al., 2024). The clinical contribution of these mechanisms in perimenopausal and postmenopausal women remains uncertain. Within the proposed oral-genitourinary model, periodontal inflammation might influence urinary susceptibility through circulating signals or through the intestinal and vaginal processes described above. Neither route has been established as a complete causal pathway in this population (**Figure 5**).

**Figure 5:**
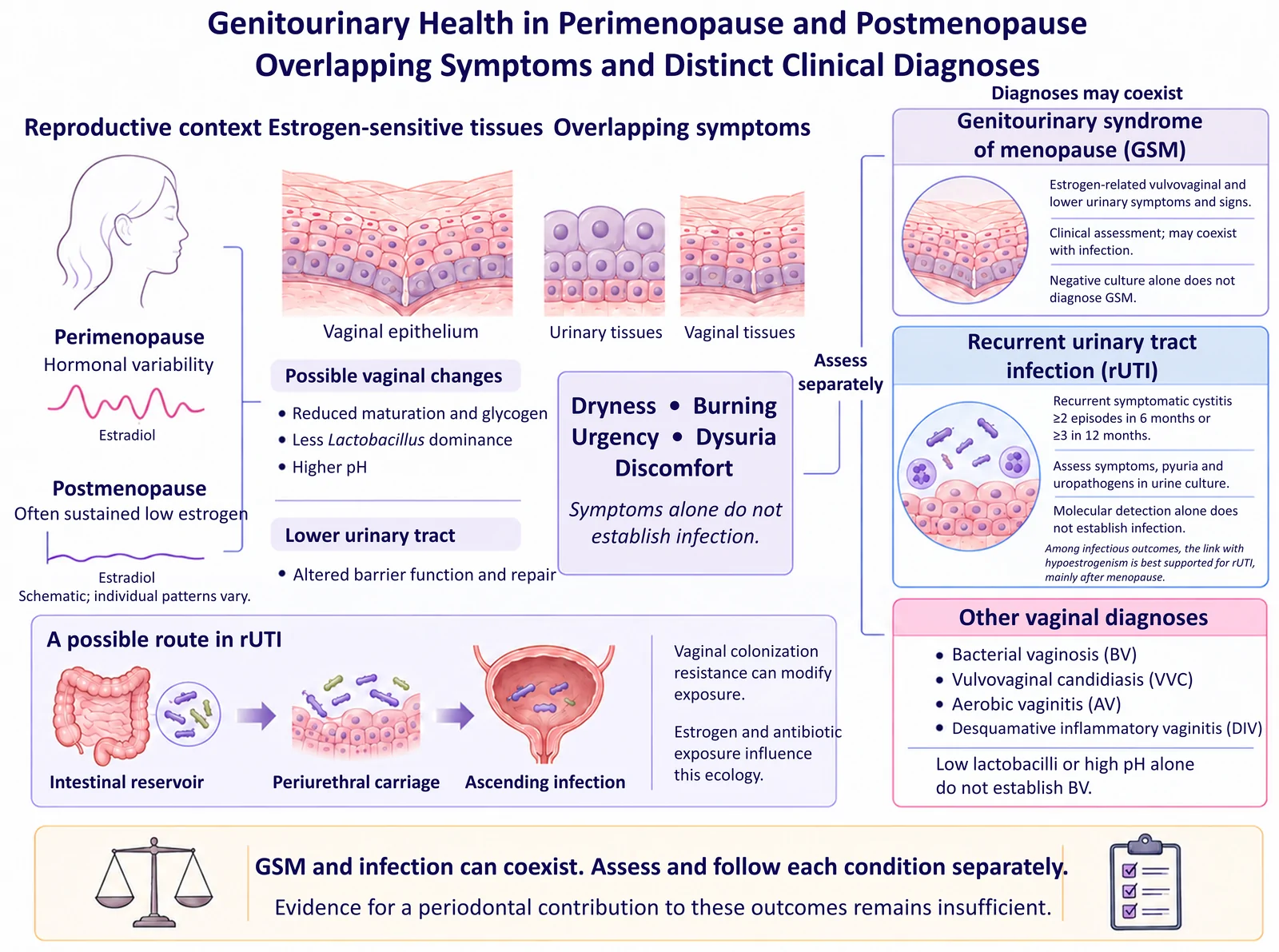
Genitourinary health in perimenopause and postmenopause. Estrogen-related changes in vaginal and lower urinary tissues provide a shared context for symptoms that require separate clinical assessment. GSM is a clinical syndrome and may coexist with infection; a negative culture alone does not diagnose it. Recurrent symptomatic cystitis is assessed through symptoms and appropriate urinary findings, including pyuria and urine culture. Molecular detection alone does not establish infection. BV, VVC, AV and DIV remain distinct diagnoses, and low lactobacilli or high vaginal pH alone do not establish BV. The lower inset depicts one possible intestinal-reservoir and ascending-infection route, modified by vaginal colonization resistance and estrogen or antibiotic exposure. It does not establish an obligatory transmission sequence. Among infectious outcomes, the hypoestrogenism relationship is best supported for rUTI, mainly in postmenopausal women. The proposed periodontal contribution remains unproven. Hormone curves and tissue illustrations are schematic. AV, aerobic vaginitis; BV, bacterial vaginosis; DIV, desquamative inflammatory vaginitis; GSM, genitourinary syndrome of menopause; rUTI, recurrent urinary tract infection; VVC, vulvovaginal candidiasis.

### 5.3 Vaginal disorders in perimenopause and postmenopause

Bacterial vaginosis (BV) and vulvovaginal candidiasis (VVC) require distinct interpretation during perimenopause and postmenopause. Hypoestrogenism reduces lactobacilli and raises vaginal pH, reproducing features used in Amsel and Nugent criteria; these findings alone are therefore insufficient to diagnose BV in a hypoestrogenic woman. Both approaches were developed mainly in reproductive-age populations, and their interpretation after menopause requires caution. Reported BV prevalence also varies with the population, estrogen exposure, and diagnostic method (Muzny et al., 2023; Van Gerwen et al., 2023). Assessment should consider epithelial maturation, discharge characteristics, clue cells, and the degree of inflammation. Molecular tests may provide additional information, but validation in postmenopausal populations remains limited.

Human profiling and experimental studies link BV-associated microbial and metabolic features to epithelial dysfunction (Berard et al., 2023). A 2025 randomized trial in premenopausal women with BV and an ongoing male partner found less recurrence when partners were also treated (Vodstrcil et al., 2025). This supports a role for partner-associated microbial exposure in the studied population; its applicability to postmenopausal women requires separate evaluation.

VVC follows a different pattern. Recurrent disease is associated with an estrogenized vaginal environment and factors such as diabetes, antibiotic exposure, immunosuppression, and host susceptibility, rather than with estrogen deficiency alone (Donders et al., 2022). A referral-clinic study of postmenopausal women found clinical VVC more frequently among hormone therapy users than nonusers. This observation is relevant to differences in estrogen exposure, although it should not be interpreted as a population prevalence estimate (Fischer and Bradford, 2011). Because Candida detection may represent colonization, recurrent or complicated cases require clinical correlation and fungal culture or PCR, particularly to identify *non-albicans* species; susceptibility testing may be needed when resistance is suspected (Nyirjesy et al., 2022).

Aerobic vaginitis (AV) adds a different inflammatory phenotype. Microscopy identifies reduced lactobacilli together with cocci or coarse bacilli, inflammatory cells, and varying epithelial immaturity. Associated organisms include *E. coli* and group B streptococci, but bacterial detection alone does not define the condition. The original observational study distinguished AV from BV through its inflammatory and epithelial features (Donders et al., 2002).

Desquamative inflammatory vaginitis (DIV) presents with vaginal inflammation and purulent discharge, commonly accompanied by numerous leukocytes and parabasal cells on microscopy. Diagnosis requires assessment for other causes of vaginal inflammation. In a referral series of 98 women with DIV, the mean age was 48.6 years and half were postmenopausal. This supports its relevance around and after menopause, but the selected clinical population does not establish its prevalence in the wider population (Sobel et al., 2011).

AV and DIV can overlap, and inflammatory patterns resembling DIV may coexist with hypoestrogenic epithelial changes. Parabasal cells indicate epithelial immaturity and are not specific evidence of infection. The clinical distinction therefore depends on the combination of symptoms, examination, epithelial maturation, inflammation, and appropriate microbiological findings. Across reproductive aging, reduced lactobacilli and higher vaginal pH should be interpreted in this context rather than assigned automatically to BV, VVC, or recurrent infection.

### 5.4 Systemic inflammation as a potential amplifier of genitourinary vulnerability

As discussed in Section 2.1, longitudinal SWAN data show heterogeneous hs-CRP and IL-6 trajectories around the final menstrual period, with increases confined to some groups. Age, adiposity, and metabolic health also influence the systemic context in which menopausal changes occur (Mehta and Manson, 2024). These observations do not support treating either perimenopause or postmenopause as a uniform inflammatory state.

Studies of the human female genital tract describe changes in epithelial and immune function with reproductive aging, with effects that differ between anatomical sites (Rodriguez-Garcia et al., 2021). Evidence concerning impaired bladder repair and altered immunity with aging also includes substantial experimental work (Ligon et al., 2023). In mouse models, suburothelial perivascular macrophages help contain invading uropathogens and coordinate local defenses (Li et al., 2025). Macrophages, neutrophils, epithelial antimicrobial programs, and inflammatory cytokines are essential for pathogen clearance, but excessive or poorly resolved responses can also promote tissue injury (Kuhn et al., 2023). Systemic inflammatory exposure could modify these responses, although the cited findings do not demonstrate a periodontal effect in women.

The postmenopausal metagenomic findings discussed above are observational and cannot establish whether microbial differences precede recurrence (Neugent et al., 2022). The urothelial memory experiments concern previous local infection in mice (Russell et al., 2023), whereas the vaginal mTOR study examines microbial and metabolic contributions to epithelial dysfunction through human profiling and experimental analyses (Berard et al., 2023). These studies support different components of mucosal susceptibility; they do not establish a common mechanism linking periodontal inflammation to all genitourinary outcomes.

Systemic inflammation should therefore be viewed as a potential amplifier rather than a sufficient cause of recurrence. Testing the proposed periodontal contribution will require measurements of periodontal inflammatory burden and systemic markers before independently assessed genitourinary outcomes. Relevant variables include age and reproductive stage, adiposity, diabetes, smoking, antibiotic and hormone exposure, sexual activity, urinary dysfunction, previous UTI, healthcare use, and previous periodontal treatment. Their roles as shared causes, mediators, or consequences should be considered according to their timing.

The clinical evidence connecting hypoestrogenism with rUTI is stronger than the evidence for corresponding effects on BV, VVC, AV, or DIV. A periodontal contribution to rUTI remains uncertain, and the evidence reviewed here does not establish periodontal amplification of GSM symptoms or any individual vaginal disorder. These outcomes should remain separate when the proposed axis is evaluated.

## 6 Systemic inflammation as a potential link between periodontal inflammation and genitourinary vulnerability

### 6.1 Systemic influences on bladder barrier function and immunity

Differences in urinary symptoms and infection recurrence during perimenopause and postmenopause may reflect variation in metabolic health, immune responses, and previous infection, as well as estrogen exposure. These observations provide a rationale for examining systemic influences on bladder susceptibility. They do not establish that circulating inflammation causes infection or that persistent urinary symptoms indicate bacterial recurrence.

The bladder urothelium is a dynamic antimicrobial barrier whose umbrella cells, tight junctions, uroplakins, glycocalyx, and progenitors restrict bacterial entry (Jafari and Rohn, 2022). Diabetes provides an example of how systemic metabolic conditions can affect this barrier. Hyperglycemia suppresses the antimicrobial peptide psoriasin, alters cytoskeletal and junctional proteins, and increases Escherichia coli burden in studies combining human samples, cell experiments, and diabetic mice (Mohanty et al., 2022). In a separate experimental study, urothelial insulin-receptor deletion increased permeability and reduced antimicrobial peptide expression, linking impaired metabolic signaling to weakened bladder defense (Schwartz et al., 2024). These findings support a role for metabolic signaling in local defense. They do not isolate systemic inflammation as the mediator or demonstrate a periodontal contribution.

Evidence concerning aging and bladder immune memory also comes largely from experimental models. In aged mice, urothelial senescence, oxidative stress, and NLRP3, gasdermin D, and IL-1β signaling have been linked to pyroptotic epithelial shedding and infection susceptibility (Joshi et al., 2024). In mouse infection models, bladder tissue-resident memory T cells, rather than circulating memory populations, mediate enhanced protection against repeat UTI (Rousseau et al., 2023). Other work implicates mast-cell activity and sensory-nerve sprouting in persistent bladder pain and dysfunction after recurrent infection (Hayes et al., 2024). These processes concern local infection history and tissue responses; they should not be attributed to periodontal inflammation without direct evidence.

Systemic inflammation may therefore modify urothelial resilience and immune-response thresholds. Direct longitudinal evidence linking circulating inflammation during perimenopause or postmenopause to microbiologically confirmed recurrent UTI remains insufficient. Future studies must distinguish hormonal effects from adiposity, glycemic status, aging, medication exposure, and previous infection.

### 6.2 Myeloid activation and persistent immune reprogramming

Chronic inflammation may influence subsequent immune responses through effects on hematopoietic stem and progenitor cells. As discussed in Section 4.3, central trained immunity should be reserved for persistent functional changes supported by metabolic or epigenetic remodeling, rather than inferred from elevated cytokines or activated circulating cells alone (Ochando et al., 2023). Human studies of severe viral infection have identified persistent epigenetic changes in innate immune cells and their progenitors (Cheong et al., 2023). These findings establish a mechanism in a different disease setting. They do not show that hormonal changes during perimenopause or postmenopause, or periodontal inflammation, induce comparable changes that affect urinary outcomes.

Within the bladder, recruited myeloid cells support bacterial clearance but may also amplify epithelial exfoliation, oxidative injury, and inflammatory signaling (Bowyer et al., 2022). The consequences depend on the timing and balance of antimicrobial defense, tissue injury, and repair (Timm et al., 2025). A heightened inflammatory response is therefore not necessarily protective, but neither does it establish persistent immune training.

In a mouse model, unresolved UTI sustained systemic inflammatory and granulocyte responses and altered distant mammary tissue (Henry et al., 2024). This observation also makes reverse causation relevant: systemic immune activation measured during recurrence may be a consequence of urinary infection. Demonstrating a periodontal contribution would require measurements before infection and evidence that the proposed immune changes precede the urinary outcome. Local urothelial memory, bladder-resident immune memory, and central myeloid reprogramming should remain distinct mechanisms.

### 6.3 Systemic inflammatory conditions and genital mucosal responses

Systemic inflammatory conditions provide indirect evidence that host physiology beyond the genital tract can influence local mucosal responses. Their relevance to the proposed periodontal pathway requires caution because metabolic changes, endocrine effects, disease-specific mechanisms, and treatment may contribute simultaneously. Hypoestrogenism, periodontal inflammatory burden, and local genital challenges are considered here as interacting factors, without assigning them to a fixed sequence of first and second hits.

An observational study found that women with obesity had less vaginal *Lactobacillus* dominance, greater microbial diversity, and higher local concentrations of several inflammatory mediators, including IL-1β, IL-6, and IL-8. The bariatric-surgery subgroup did not show a significant overall change in vaginal microbial composition during follow-up (Raglan et al., 2021). These findings show an association with systemic metabolic status, but do not establish inflammatory mediation or a reversible causal pathway.

A case-control study of primary Sjögren’s syndrome identified vaginal inflammatory and vascular abnormalities in women who were not postmenopausal (van Nimwegen et al., 2020). In another case-control study, women with systemic lupus erythematosus had altered vaginal microbial communities that were associated with systemic immunological measures, including complement levels (Ling et al., 2023). These observations concern specific autoimmune diseases and cannot be assumed to represent the effects of low-grade periodontal inflammation.

Human genital-tract profiling has also associated elevated inflammatory cytokines with protease activity, differences in barrier proteins, and immune-cell recruitment (Arnold et al., 2016). However, systemic inflammatory disease does not invariably produce vaginal dysbiosis: menopausal status influenced vaginal communities more strongly than inflammatory bowel disease diagnosis or activity in one cohort (Bar et al., 2023).

Systemic inflammation should therefore be regarded as a possible, context-dependent modifier of genital mucosal responses. The evidence discussed here does not establish periodontal amplification of GSM symptoms, BV, VVC, AV, or DIV. These outcomes require separate clinical definitions and prospective assessment in perimenopausal and postmenopausal women.

## 7 A proposed estrogen-sensitive oral-systemic-genitourinary axis

### 7.1 Three interacting components

The evidence reviewed above supports consideration of a proposed oral-systemic-genitourinary relationship during perimenopause and postmenopause. The model comprises three interacting components: a hypoestrogenic background that may reduce mucosal resilience, periodontal inflammatory burden as a potential amplifier, and a local genitourinary microbial or inflammatory challenge. These components need not occur in a fixed order, and their relative contributions may differ among women and clinical outcomes. Broader research on the periodontal-female reproductive axis provides additional context, although the cited scoping review concerns infertility and does not directly establish this pathway in women with recurrent genitourinary disease (Mujica et al., 2026) (**Figure 6**).

**Figure 6:**
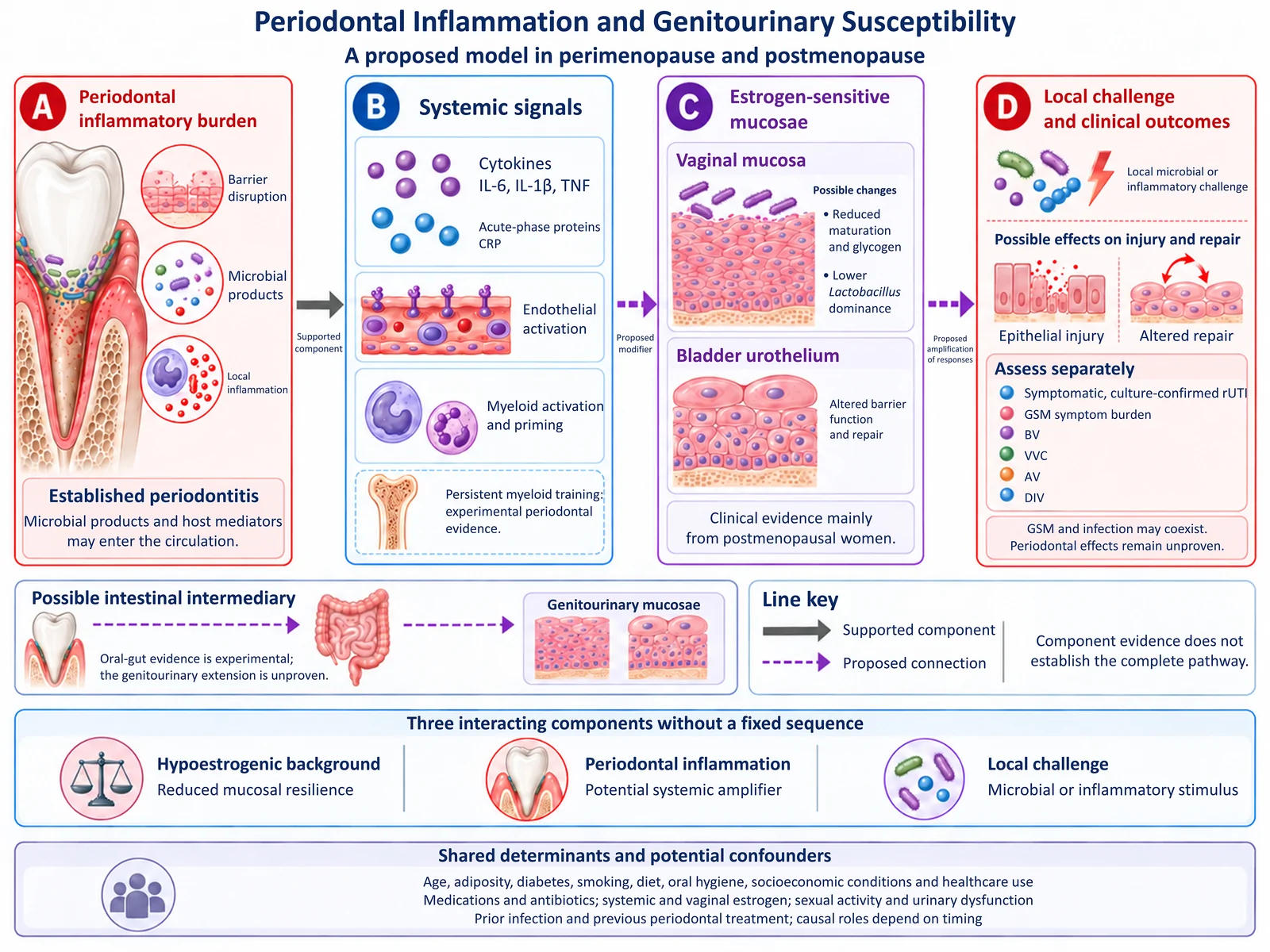
A proposed model of periodontal and genitourinary interactions. **(A)** Periodontal inflammatory burden. **(B)** Systemic signals. **(C)** Estrogen-sensitive mucosae, showing the vaginal mucosa and bladder urothelium separately. **(D)** Local challenge and clinical outcomes. The framework comprises a hypoestrogenic susceptibility background, periodontal inflammatory burden as a potential systemic amplifier, and a local microbial or inflammatory challenge. These components do not require a fixed sequence. Periodontal inflammation may contribute to circulating signals, with cytokines distinguished from acute-phase proteins. Persistent myeloid training is identified as experimental periodontal evidence, not an established mechanism of urinary recurrence in women. Vaginal squamous epithelium and bladder urothelium are shown separately; the Lactobacillus label applies to vaginal ecology. The intestinal inset represents a candidate intermediary, with experimental support for oral-gut effects but no established complete genitourinary pathway. Solid arrows identify supported component relationships and dashed arrows identify proposed connections. Their relative contributions to clinical outcomes remain uncertain. Urinary recurrence, GSM symptoms and individual vaginal diagnoses require separate assessment. Shared determinants may influence several compartments, and their causal roles depend on timing. CRP, C-reactive protein; IL, interleukin; TNF, tumor necrosis factor. AV, aerobic vaginitis; BV, bacterial vaginosis; DIV, desquamative inflammatory vaginitis; GSM, genitourinary syndrome of menopause; rUTI, recurrent urinary tract infection; VVC, vulvovaginal candidiasis.

The proposed periodontal exposure is the extent and persistence of inflammation in periodontal tissues, assessed alongside a clinical diagnosis of periodontitis. Microbial dysbiosis describes an ecological disturbance and should not substitute for measurement of tissue inflammation. Ulcerated periodontal tissues can repeatedly release bacterial products and host inflammatory mediators, contributing to systemic inflammatory burden (Tattar et al., 2025). The proposed connection does not require stable colonization of the urinary or vaginal tract by periodontal pathogens. Circulating signals could influence mucosal responses directly, while changes in intestinal ecology or immunity could provide an additional route through vaginal and periurethral reservoirs to bladder susceptibility. The latter route brings together the oral-gut evidence in Section 4.3 and the gut-vagina-bladder processes in Section 5.2, but remains an extrapolation. Human evidence for periodontal effects on circulating inflammatory markers is more direct than evidence for the complete intestinal and vaginal pathway; their relative contributions to genitourinary disease have not been compared.

### 7.2 Indirect evidence and the limits of the clinical findings

In germ-free mice, administration of periodontitis-associated human oral microbiota altered intestinal microbial composition, hepatic transcriptional programs, and circulating metabolites compared with health-associated oral microbiota (Yamazaki et al., 2022). This experiment supports an indirect systemic effect in a selected model; it did not assess genitourinary outcomes.

Salivary microbiota obtained from patients with periodontitis aggravated systemic osteoclastogenesis and bone loss in ovariectomized mice by remodeling the gut microbiota and suppressing tryptophan metabolism; restoration of the microbial metabolite indole-3-lactic acid attenuated this phenotype (Wang et al., 2026). This provides a mechanistic precedent for distant effects in an estrogen-deficient host. Ovariectomy does not reproduce the hormonal variability of perimenopause, and a skeletal outcome cannot establish a corresponding effect on urinary or vaginal disease.

The most direct clinical study was an exploratory randomized trial whose 30 female participants were distributed among control, oral-hygiene, and periodontal-treatment groups. It reported improved periodontal measures and fewer positive urine cultures after treatment, but did not distinguish symptomatic infection from asymptomatic bacteriuria. Menopausal status was not analyzed, follow-up was short, and inflammatory or multisite microbial mediators were not assessed. Registration was retrospective, and the sample-size calculation concerned the male prostate-specific antigen outcome. Antibiotic use was included in adjusted analyses, but concurrent urological management was insufficiently characterized to establish mediation (Castro et al., 2026). This result does not establish prevention of symptomatic rUTI or the proposed inflammatory pathway.

Peripheral reproductive evidence is also indirect. A prospective endometriosis cohort identified differences across oral, vaginal, and stool microbial communities (Hicks et al., 2025). while a translational study implicated endometrial *Fusobacterium* in experimental lesion development (Muraoka et al., 2023). Neither demonstrated a periodontal source, transmission to the reproductive tract, or an effect on the genitourinary outcomes considered here.

### 7.3 Testing the proposed relationship

Longitudinal studies should measure periodontal inflammation and systemic signals before independently assessed genitourinary outcomes. Sampling must account for the transient inflammatory response following periodontal instrumentation and subsequent tissue healing (Mainas et al., 2022). Randomized periodontal-treatment studies in other clinical populations demonstrate that oral inflammation and some systemic outcomes can be modified, but do not establish an effect on urinary or vaginal recurrence (D’Aiuto et al., 2018).

Serial sampling should include subgingival plaque, blood, stool, vaginal or periurethral samples, and urine in appropriately staged perimenopausal and postmenopausal participants. Genus-level overlap between anatomical sites is insufficient to demonstrate dissemination or its direction. Strain-resolved analyses can assess relatedness, but require temporal and epidemiological information to support transmission (Valles-Colomer et al., 2023). Microbial data should be integrated with estrogen exposure, systemic inflammatory markers, myeloid phenotypes, epithelial injury, antibiotic use, and symptomatic, culture-confirmed urinary recurrence, using standardized reporting and contamination controls (Mirzayi et al., 2021).

Potential shared determinants include age, reproductive stage, adiposity, diabetes, smoking, diet, oral hygiene, socioeconomic conditions, healthcare use, and medication exposure. Sexual activity, urinary dysfunction, previous infection, systemic or vaginal estrogen, antibiotic use, and previous periodontal treatment should also be recorded. Their treatment as confounders, mediators, or consequences should depend on the prespecified causal question and the timing of measurement.

A factorial trial could test whether reducing periodontal inflammation adds benefit to vaginal estrogen in eligible perimenopausal and postmenopausal women with periodontitis and rUTI. The four groups would receive periodontal treatment, vaginal estrogen, both interventions, or usual care within an ethically appropriate design that preserves indicated treatment. The primary outcome should be the rate of symptomatic, culture-confirmed urinary recurrences over a prespecified follow-up period. Secondary outcomes should include periodontal inflammatory burden, systemic inflammatory markers, urogenital microbial composition, antibiotic use, and patient-reported urinary and GSM symptoms. BV, VVC, AV, and DIV should be diagnosed and analyzed separately if included. The study should estimate the effects of each intervention and their interaction; combined benefit should not be assumed in advance.

### 7.4 What is established, what is plausible, and what remains unproven

Established evidence supports a contribution of periodontitis to systemic inflammatory burden and a role for reduced estrogen exposure in genitourinary changes. Vaginal estrogen has clinical evidence for preventing rUTI in eligible women, with much of the trial evidence derived from postmenopausal populations. These findings support individual components of the proposed model.

It is biologically plausible that periodontal inflammation could modify genitourinary responses through circulating inflammatory signals or intermediate intestinal and vaginal processes. Persistent myeloid reprogramming is a candidate mechanism supported in other settings, but has not been demonstrated as the link in this population.

What remains unproven is whether periodontal inflammation increases symptomatic rUTI during perimenopause or postmenopause, whether periodontal treatment reduces that risk, and which mechanisms would mediate any benefit. Evidence is still less direct for periodontal effects on GSM symptoms, BV, VVC, AV, or DIV. Periodontal inflammation is a modifiable exposure, but its proposed contribution to these clinical outcomes remains a hypothesis. **Table 1** summarizes the evidence and its limitations.

**Table 1:** Evidence supporting the proposed relationship.

Relationship	Population and design	Principal finding	Relevance and limitation	Directness
Reproductive aging and oral ecology	Women across reproductive stages; mainly observational studies (LaMonte et al., 2021; Soliman et al., 2022; Yakar et al., 2025; J Rus et al., 2026)	Microbial and periodontal measures vary with hormonal exposure and clinical context.	Sampling, age, medication and past tissue damage limit inference; no uniform signature or complete pathway demonstrated.	Component evidence
Periodontal inflammation and systemic markers	Human treatment studies and syntheses (Orlandi et al., 2022; da Silva Barbirato et al., 2024; Matsuda et al., 2024)	Periodontal treatment can lower systemic inflammatory markers.	Supports a modifiable inflammatory exposure; biomarker changes do not establish prevention of urinary or vaginal disease.	Clinical component
Persistent myeloid reprogramming	Experimental periodontitis, human progenitor experiments, severe viral infection (Li et al., 2022; Cheong et al., 2023; Flores-Gomez et al., 2024)	Selected models show lasting changes in progenitors or innate responses.	Different stimuli and outcomes; not evidence of periodontal myeloid training causing rUTI in peri- or postmenopausal women.	Indirect mechanism
Oral-gut and distant metabolic effects	Mouse transfer and disease models, including ovariectomized mice (Kitamoto et al., 2020; Bao et al., 2022; Yamazaki et al., 2022; Wang et al., 2026)	Oral communities can alter intestinal ecology, inflammation or distant metabolism.	Model-specific colitis, metabolic or skeletal outcomes; complete oral-gut-vagina-bladder pathway not tested.	Experimental component
Metabolic state and bladder defense	Human samples, urothelial cells and mice (Mohanty et al., 2022; Schwartz et al., 2024)	Glucose and insulin signaling affect epithelial and antimicrobial defenses.	Does not isolate circulating inflammation or periodontal disease as the initiating exposure.	Indirect mechanism
Systemic disease and vaginal responses	Obesity, autoimmune disease and IBD studies; genital-tract profiling (Arnold et al., 2016; van Nimwegen et al., 2020; Raglan et al., 2021; Bar et al., 2023; Ling et al., 2023)	Some systemic conditions accompany vaginal inflammatory or microbial differences.	Disease-specific mechanisms and treatments matter; findings are not uniform and do not establish periodontal effects.	Indirect association
GSM	Clinical literature on reproductive aging (Sarmento et al., 2021; Waetjen et al., 2023; Powell et al., 2024; Kaufman et al., 2025)	Reduced estrogen exposure contributes to vulvovaginal and lower urinary tract symptoms and signs.	GSM can coexist with infection; periodontal amplification of GSM symptoms has not been demonstrated.	Clinical phenotype; periodontal link unproven
rUTI and vaginal estrogen	Postmenopausal randomized trial (Ferrante et al., 2021); 2025 AUA/CUA/SUFU guideline (Ackerman et al., 2026)	Clinical evidence supports prevention in eligible women.	Establishes a local hormonal intervention; does not test periodontal treatment or all proposed mediators.	Clinical component
rUTI and intestinal or vaginal ecology	Longitudinal cohort (Worby et al., 2022); postmenopausal cross-sectional study (Horseman et al., 2026)	rUTI history is associated with intestinal and urogenital ecological differences.	Antibiotics and shared host factors complicate directionality; concordance does not establish strain transmission.	Observational component
Periodontal treatment and urinary findings	Exploratory randomized study including 30 women (Castro et al., 2026)	Fewer positive cultures reported after treatment.	Symptomatic recurrence not separately established; see Section 7.2 for design and mediation limits.	Closest direct study; inconclusive
BV	Clinical and profiling studies; premenopausal partner-treatment trial (Berard et al., 2023; Muzny et al., 2023; Van Gerwen et al., 2023; Vodstrcil et al., 2025)	BV-related ecology and recurrence depend on host and partner context.	Higher pH and reduced lactobacilli overlap with hypoestrogenic changes; no established periodontal effect.	Phenotype evidence; periodontal link unproven
VVC	Clinical literature (Donders et al., 2022; Nyirjesy et al., 2022); postmenopausal hormone-therapy referral study (Fischer and Bradford, 2011)	Estrogen exposure and other host factors influence clinical VVC.	Candida detection may be colonization; referral findings are not population prevalence; no established periodontal effect.	Phenotype evidence; periodontal link unproven
AV	Original observational microscopy study (Donders et al., 2002)	Inflammatory and epithelial features distinguish AV from BV.	Bacterial detection alone is insufficient; no direct periodontal evidence identified in the reviewed material.	Phenotype evidence; periodontal link unproven
DIV	Referral series of 98 women (Sobel et al., 2011)	Inflammatory vaginitis occurs around and after menopause.	Selected clinical population; epithelial immaturity is nonspecific; periodontal amplification not established.	Phenotype evidence; periodontal link unproven

AV, aerobic vaginitis; BV, bacterial vaginosis; DIV, desquamative inflammatory vaginitis; GSM, genitourinary syndrome of menopause; IBD, inflammatory bowel disease; rUTI, recurrent urinary tract infection; VVC, vulvovaginal candidiasis.

## 8 Literature search and evidence interpretation

This article is a hypothesis-generating narrative review. During revision, supplementary targeted searches of PubMed were conducted on 8 September 2026 to examine literature relevant to the existing synthesis. English-language records published through that date were considered, without restricting the search to recent publication years. Search terms were combined by topic and included oral microbiome, oral microbiota, biofilm, periodontitis, periodontal treatment, systemic inflammation, C-reactive protein, perimenopause, menopause, postmenopause, genitourinary syndrome, recurrent urinary tract infection, vaginal estrogen, bacterial vaginosis, candidiasis, aerobic vaginitis, desquamative inflammatory vaginitis, oral-gut, gut-vagina, gut-bladder, trained immunity, myeloid priming, and epithelial memory. Additional focused searches identified randomized trials of periodontal treatment and estrogen therapy relevant to systemic inflammatory or urinary outcomes.

Selected titles and abstracts were examined for relevance to the proposed model, with attention to the study population, design, outcome definitions, and applicability to perimenopausal or postmenopausal women. In the synthesis, human intervention and longitudinal studies carry greater weight for clinical interpretation; cross-sectional studies describe associations, while animal and cellular studies inform biological plausibility within their tested settings. Reviews provide background and context, and clinical guidelines inform definitions and management statements. Earlier foundational studies remain relevant where they establish key concepts or diagnostic distinctions. Conflicting findings, shared determinants, and limitations of extrapolation are considered alongside supportive evidence. Evidence for individual components is distinguished from evidence directly testing the complete periodontal-genitourinary relationship. The supplementary searches were selective and non-exhaustive; no formal risk-of-bias scoring or quantitative pooling was undertaken.
